# Muscle Fatty Acid Content in Selected Freshwater Fish from Bukit Merah Reservoir, Perak, Malaysia

**DOI:** 10.21315/tlsr2018.29.2.8

**Published:** 2018-07-06

**Authors:** Annette Jaya-Ram, Farhana Fuad, Mohd. Shafiq Zakeyuddin, Amir Shah Ruddin Md. Sah

**Affiliations:** 1 Centre for Marine and Coastal Studies, Universiti Sains Malaysia, 11800 USM Pulau Pinang, Malaysia; 2 School of Biological Sciences, Universiti Sains Malaysia, 11800 USM Pulau Pinang, Malaysia

**Keywords:** Polyunsaturated Fatty Acids, Inland Fisheries, Freshwater Fish, Bukit Merah Reservoir, Asid Lemak Politaktepu, Perikanan Daratan, Ikan Air Tawar, Reservoir Bukit Merah

## Abstract

One of the oldest reservoirs in Peninsular Malaysia, Bukit Merah Reservoir, is a place in which locals participate in fishing activities. Inland fisheries are important to individuals, society and the environment; whereby they generate a source of income and food security. It is essential to gauge the nutrition value of fish caught in this location as food source, especially in terms of fatty acid composition, to better demonstrate its potential towards the betterment of human health and general well-being. From an initial list of 47 fish species available in Bukit Merah Reservoir, a total of seven edible freshwater fish species were identified, namely tinfoil barb (*Barbonymus schwanenfeldii*), Javanese barb (*Barbonymus gonionotus*), hampala barb (*Hampala macrolepidota*), beardless barb (*Cyclocheilichthys apogon*), glassfish (*Oxygaster anomalura*), striped snakehead (*Channa striata*) and horseface loach (*Acantopsis dialuzona*), and muscle fatty acid content was analysed to determine their nutritional value. Muscle of cyprinid fish contained substantial amount of omega-3 long-chain polyunsaturated fatty acids (LC-PUFA) compared to fish from Channidae and Cobitidae families. Javanese and tinfoil barbs muscle recorded the highest levels of combined eicosapentaenoic acid (EPA) and docosahexaenoic acid (DHA) indicating the highest nutritional value comparatively. Unexpectedly, the striped snakehead, a predatory carnivore, contained lower levels of LC-PUFA compared to detrivorous/herbivorous fishes. This further justifies that the omega-3 polyunsaturated fatty acids (PUFA) content in fish muscle varies according to their feeding habits. Even though it has been recommended that marine fish be consumed to improve health to a certain extent, there still are benefits of consuming freshwater fish, as there are several species which contain considerable amounts of beneficial omega-3 PUFA.

## Abstract (ms)

Reservoir Bukit Merah ialah salah satu takungan air yang tertua di Semenanjung Malaysia dan menjadi lokasi masyarakat tempatan menjalankan kegiatan penangkapan ikan. Aktiviti perikanan daratan penting bagi individu, masyarakat dan juga persekitaran kerana kegiatan ini dapat menjana sumber pendapatan dan memberi jaminan makanan. Nilai nutrisi ikan yang ditangkap di lokasi ini, terutamanya kandungan asid lemak, penting untuk diketahui, agar potensi terhadap peningkatan kesihatan manusia secara am dapat didemonstrasikan. Daripada senarai asal yang mengandungi 47 spesies ikan yang dijumpai di Reservoir Bukit Merah, sejumlah tujuh spesies ikan air tawar yang boleh dimakan telah dikenal pasti, iaitu lampam sungai (*Barbonymus schwanenfeldii*), lampam jawa (*Barbonymus gonionotus*), sebarau (*Hampala macrolepidota*), temperas (*Cyclocheilichthys apogon*), lalang (*Oxygaster anomalura*), haruan (*Channa striata*) dan pasir (*Acantopsis dialuzona*), dan kandungan asid lemak otot telah dianalisa untuk menentukan nilai nutrisi masing-masing. Otot ikan siprinid mengandungi lebih jumlah asid lemak politaktepu rantai panjang omega-3 (LC-PUFA) berbanding ikan daripada famili Channidae dan Cobitidae. Kandungan gabungan asid eikosapentaenoik (EPA) dan dokosaheksaenoik (DHA) yang tertinggi telah direkod pada otot ikan lampam jawa dan lampam sungai, ini menunjukkan kandungan nutrisi tertinggi berbanding spesies lain. Ikan haruan, iaitu sejenis pemangsa bersifat karnivor, mengandungi tahap LC-PUFA yang rendah berbanding ikan yang bersifat detrivor/herbivor. Hal ini menunjukkan bahawa kandungan asid lemak politaktepu (PUFA) omega-3 dalam otot ikan berbeza dan mengikut tabiat pemakanan mereka. Walaupun terdapat saranan bahawa memakan ikan laut dapat meningkatkan kesihatan kepada suatu tahap, namun demikian, masih terdapat manfaat daripada memakan ikan air tawar kerana terdapat beberapa spesies yang mengandungi PUFA omega-3 yang tinggi.

## INTRODUCTION

There are several benefits of incorporating polyunsaturated fatty acids (PUFA) in human diet, as they are capable of reducing atherosclerosis, lowering blood pressure and diminishing depression. Recommendations have also been made to replace saturated and trans-fatty acids with PUFA to assist in cardiovascular disease prevention (Erkkila *et al.* 2008). Plants, oil seeds and fatty fish consumption can contribute towards obtaining natural sources of PUFA. PUFA such as α-linolenic acid (ALA, 18:3n-3) and linoleic acid (LA, 18:2n-6) are essential dietary components for all vertebrates, as they are unable to be synthesised *de novo* from monounsaturated fatty acids (MUFA) (Bell & Tocher 2009). Long-chain polyunsaturated fatty acids (LC-PUFA) such as eicosapentaenoic acid (EPA, 20:5n-3), docosahexaenoic acid (DHA, 22:6n-3) and arachidonic acid (ARA, 20:4n-6) are usually found in abundance in fish. It has been shown that LC-PUFA contributed beneficially towards diseases such as inflammatory and cardiovascular disorders, neural development and neurological conditions (Calder 2006; Horrobin 1993; Simopoulos 1991). LC-PUFA such as ARA and EPA are precursors of docosanoids and eicosanoids, a group of fatty acids which are vital components in cell membranes and also mediate and control several cellular activities (Ahlgren *et al.* 2009).

Synthesis of LC-PUFA from shorter chained fatty acid precursors in humans are limited, therefore consuming fish has become a significant source of dietary LC-PUFA (Leaver *et al.* 2008). The requirement of fish for consumption does not only cater to the increasing human population, but also to fulfil the nutritional requirements and reap the health benefits. It was also recognised that fish were the optimal source of the LC-PUFA, and also combats several human health pathologies stemming from imbalance or a lack of these fatty acids, demand for fish remained high (de Silva *et al.* 2011). In order to satisfy the nutritional demand of health beneficial LC-PUFA, humans are heavily relying on the aquatic ecosystem. To date, the request for seafood continues to rise even though wild fisheries are being fully exploited and overharvested. Marine fish such as salmon, sea bass, cod and barramundi may have more than four times the omega-3 content of freshwater fish such as carp, and are becoming more popular after for their positive health benefits (Schipp 2008). However, this does not mean that freshwater fish aren’t captured and consumed. In Malaysia and neighbouring countries such as Thailand and Indonesia, freshwater fish consumption is high, especially in areas where inland water bodies are a main source of fish, be it for food or recreation. Based on the Annual Fisheries Statistic Report for the year 2016, inland fisheries landing is 5847.97 metric tonnes, which includes catch landing from rivers, ex mining pools, reservoirs and lakes (Department of Fisheries Malaysia 2017).

Marine fish species are generally characterised with lipids containing low levels of linolenic acid (18:3n-3, ALA) and linoleic acid (18:2n-6, LA), but having high levels of omega-3 LC-PUFA such as EPA and DHA (Özogul *et al.* 2007; Ugoala *et al.* 2008). Further comparison of marine fish with freshwater fish species reveals the higher content of the omega-6 series fatty acids in freshwater fish and higher levels of omega-3 fatty acids in marine fish (Gutierrez & da Silva 1993; Steffens 1997). However, the variation of fatty acid composition can differ vastly even within the same species sampled at different time and locations for either marine or freshwater fish species. Regardless of the environment in which the fish is sourced from, consumption of fish contributes as an important protein and essential nutrient source. Benefits of fish consumption also extends beyond omega-3 LC-PUFA content, whereby fish as a whole food provides protein and vitamins and minerals inclusive of vitamin A, D, zinc, selenium and iodine (McManus *et al*. 2011).

Reservoirs in Malaysia are predominantly constructed for irrigation, hydroelectric power generation, drinking water supply and reduce floods. However, there are some communities which rely on lakes, reservoirs and the associated rivers for their livelihoods (Ambak & Jalal 2006). Bukit Merah Reservoir is one of the oldest man-made reservoirs, was constructed in 1902 and is located in the state of Perak in Peninsular Malaysia (Mohd. Shafiq *et al.* 2014). It functions mainly as water irrigation for paddy fields in the locality of north Kerian rice agroecosystem. This reservoir is also frequented by local people who capture available fish either to be sold as food source or for recreational activities. Studies conducted in Bukit Merah Reservoir encompassed the rich diversity of freshwater fish present, whereby early surveys dates back to the 1930s. The most recent checklist available in Bukit Merah Reservoir revealed a number of 47 fish species (Mohd. Shafiq *et al.* 2014). Inland fisheries are an important source of fish to some communities which have limited access to consuming marine fish, be it geographically or economically challenged. It is also estimated that around 90% of fish from inland captures are for human consumption purposes, contrary to marine fisheries whereby a substantial amount is contributed for the production of fish meal (Welcomme *et al.* 2010). The importance and contribution of inland fisheries towards human consumption is usually overshadowed by marine fisheries comparatively because of its magnitude (Lynch *et al.* 2016). Besides this, it has also been suggested that inland fishes are more diverse than what has been estimated due to the difficulty in assessment especially in developing countries and in remote areas (Cooke & Cowx 2004). The harvest from small scale or artisanal fishing rarely affects the market economy and is usually not well documented. However, it is undeniable that the inland fisheries contribute significantly to food and economic security by provision of primary sources of animal protein, essential nutrients and income (Bartley *et al.* 2015). This is even more so to the rural poor and strengthens global food security. Better understanding of the significance of inland fisheries or small scale fishing resource is crucial as the importance of their contribution is paled in comparison with fisheries from larger water bodies.

In the array of studies conducted of fish species available in Bukit Merah Reservoir, data on the nutritional contribution towards human health or well-being in the perspective of fatty acid profiles of these fish are still lacking. It is also interesting to gauge if freshwater fish are able to supply sufficient LC-PUFA to a community which has limited access to marine fish. Thus, this study aims to evaluate the nutritional value of the fatty acids in flesh of edible fish sampled from this location.

## MATERIALS AND METHODS

### Study Area

**Figure 1:**
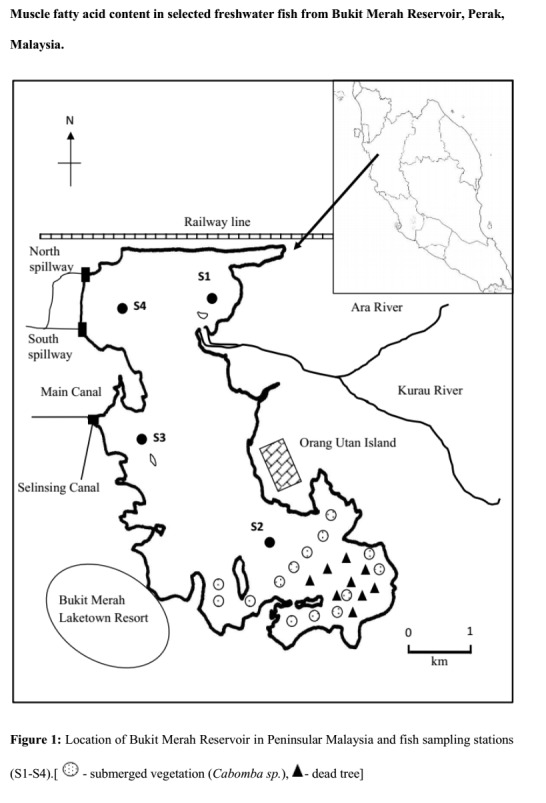
Location of Bukit Merah Reservoir in Peninsular Malaysia and fish sampling stations (S1–S4). ( 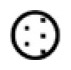 = submerged vegetation (*Ca bomba sp*.), ▲ = dead tree).

Bukit Merah Reservoir is formed by dam construction within the Kurau River and is located at 05°01′35.42″N, 100°39′42.92″E. Fish were sampled from locations as elaborated in Mohd. Shafiq *et al.* (2014) and are marked as S1–S4 in Fig. 1. For present study, edible species identified and were most abundant in availability were selected to further evaluate their nutritional content.

### Sample Collection

Experimental gill nets (2.5–13 cm mesh size) were used for fish sampling. Taxonomic keys were used to identify all fish specimens to the lowest taxation (Kottelat *et al*. 1993; Rainboth 1996; Ambak 2010). Fish were identified based on morphometric and meristic methods. Measurements of fish body lengths, number of gill rakers and number of dorsal fin spines were taken into consideration for fish identification. Freshly caught fish were subjected to cold shock by subjecting them to ice slurry. They were kept cool during transportation and immediately transferred to −80°C freezer upon arrival. Fish were dissected to obtain muscle tissues for fatty acid profile analysis. Analysis of an initial sample set of representative samples were conducted utilising three individual fish per species. Each fish was considered as a single replicate and the mean of three individual fish were utilised as final data.

### Fatty Acid Methyl Ester Extraction and Analysis by Gas Chromatography

Fish muscle tissues were subjected to total lipid extraction and fatty acid methyl esters (FAME) were prepared through methylation and transesterification using boron trifluoride in methanol (Cuniff 1997). Tissues (0.5 g–1.0 g) were mechanically homogenized in chloroform/methanol (2:1, v/v) solvent to obtain total lipid (Folch *et al.* 1957). Gas chromatograph (GC-2010, Shimadzu) equipped with a flame ionization detector and a fused silica highly polar cyanosiloxane column, SP-2380 (30 m length, 0.25 mm inner diameter, 0.20 μm film thickness; Supelco, USA) was used to separate the FAME. The temperature was programmed to increase from 100°C to 230°C at 1.5°C/min with split ratio of 1:50, and nitrogen was utilised as the carrier gas. The injector and detector temperature were set at 250°C and 260°C respectively. Individual FAME were identified based on retention times comparison from commercially available standards, 37 Component FAME Mix (Supelco) and PUFA No. 3 from Menhaden Oil (Supelco).

### Statistical Analysis

Statistical comparison between the FAME in different fish species was determined using one- way analysis of variance (ANOVA), followed by Tukey’s post hoc test at a significance level of *p* < 0.05 using the SPSS software version 20 (SPSS, USA).

## RESULTS

### Sample Collection

There were 47 species of fish identified at the Bukit Merah Reservoir (Mohd. Shafiq *et al.* 2014). Out of this list, only seven species were considered suitable for human consumption. These seven edible fish species were from three different families and were selected for muscle fatty acid content analysis. Table 1 displays the family, common and local names of the selected fish species.

### Fatty Acid Profiles

**Figure 2:**
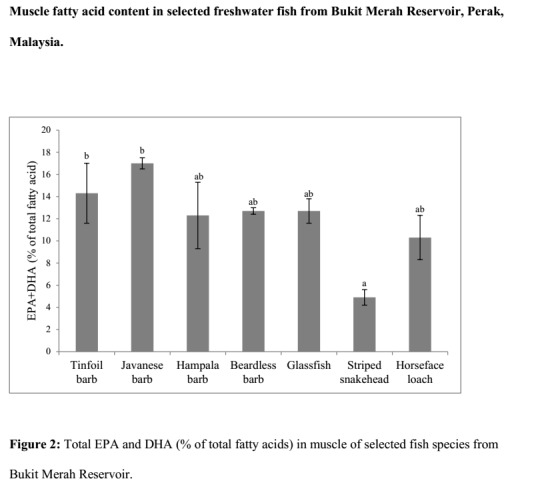
Total EPA and DHA (% of total fatty acids) in muscle of selected fish species from Bukit Merah Reservoir.

Table 2 shows the percentages of total fatty acids of the selected fish species. Species such as the horseface loach recorded the highest total saturated fatty acids (43.1%), while the highest MUFA and PUFA were recorded in the striped snakehead and beardless barb, 38.4% and 26.8% respectively. The total of EPA and DHA of these fish species are summarised in Fig. 2. The highest total of EPA and DHA was recorded in the Javanese barb followed by tinfoil barb, beardless barb/glassfish, hampala barb, horseface loach and striped snakehead.

## DISCUSSION

Seven different species of edible freshwater fish were identified for further nutritional evaluation from the initial sampling from the Bukit Merah Reservoir. Cyprinids such as tinfoil barb, Javanese barb, hampala barb, beardless barb and glassfish were the most abundant species obtained from the reservoir. Several independent factors such as physiological status, reproductive cycle, water temperature and aquatic environment have the capacity to influence the lipid composition of fish (Vasconi *et al.* 2015). The fatty acid composition of fish was also shown to be determined by their trophic position, which is either being piscivorous, herbivorous or omnivorous (Czesny *et al.* 2011).

All species of fish analysed contained high levels of the saturated fatty acid (SFA), palmitic acid (16:0), which has been indicated to be a major fatty acid in freshwater fish (Rahman *et al.* 1995). Palmitic acid obtained in this study is in the range of 21% to 27%, whereby several other studies have reported varying levels from 14% up to 35% (Gutierrez & da Silva 1993; Rahman *et al.* 1995; Cengiz *et al.* 2010). Another SFA which is also relatively ubiquitous in freshwater fish habitat is 18:0, which are reported to be present in most classes of microalgae. This is also true for monounsaturated fatty acid (MUFA) 18:1n-9 (Guedes *et al*. 2011). The occurrence of both these fatty acids is higher in the omnivorous and detrivorous fish species compared to striped snakehead and hampala barb, which are both carnivorous and predatory fish.

A notable observation from the results obtained was that the lowest EPA and DHA values were recorded in the striped snakehead fish. As a carnivorous fish, it would be assumed that these fish would be getting sufficient or high LC-PUFA levels from their natural piscine diet. Even though they mainly eat fish, they are also known to consume crustaceans, frogs, or small reptiles. In food deprived conditions, they are also capable of becoming cannibalistic towards their young (Qin & Fast 1996, 1997). On top of that, striped snakeheads have been reported to possess a series of desaturase and elongase enzymes required in the fatty acid conversion pathway of essential fatty acids such as α-linolenic acid (ALA, 18:3n-3) and linoleic acid (LA, 18:2n-6) into LC-PUFA (Kuah *et al.* 2015; 2016). In reference to other studies, fatty acid composition of striped snakeheads, especially LC-PUFA can vary drastically. Several studies stated that striped snakeheads have a good range of LC-PUFA and essential amino acid (Mat Jais *et al*. 1998; Samantaray & Mohanty 1997). Contrastingly, other studies have shown reduced amounts or no detection of EPA and DHA to 15%, and 0–19% for ARA (Zuraini *et al.* 2006; Rahman *et al.* 1995).

Tinfoil barbs are native to Malaysia, Borneo, Sumatra, Cambodia, Laos, Thailand and Vietnam. They are largely herbivorous, feeding on aquatic and terrestrial plants, or algae but are occasionally known to feed on invertebrates like insects and small fish (Gante *et al*. 2008). This fish are both popular in the ornamental fish trade and also commercial aquaculture, they are utilised in local food production (Rainboth 1996). Levels of DHA were highest in Javanese barbs and tinfoil barbs, but not significantly different compared to other cyprinids. A possible avenue of further research could be conducted in investigating the availability of short chain PUFA conversion enzymes in both mentioned species. The low levels of ALA in tinfoil barb and Javanese barb muscle, along with high levels of DHA, could potentially indicate conversion activity. High levels of shorter chain PUFA can be related to the feeding habits of fish which eats more plant matter which is rich in these essential fatty acids (Du *et al*. 2008).

From the perspective of human nutrition, it has been recommended that fish be consumed for sufficient LC-PUFA intake. In general, fish are valued for their high omega-3 fatty acid content. Omega-3 fatty acids play a role in prevention and management of cardiovascular diseases (Connor 2000). They are also important components in membrane phospholipids in tissues which are essential for proper functioning (Holub & Holub 2004). Marine fish in particular have the reputation of being a good source of omega-3 fatty acids, whilst freshwater fish have been known to be higher in omega-6 fatty acids (Ugoala *et al.* 2008). In line with this, comparison of the seven fish species muscles reveals that the LA content is indeed higher than the ALA content in all. LA is equally an important essential fatty acid in human nutrition, as it is not synthesised by the body but required for proper development.

Another omega-6 component, ARA, was richer in beardless barb, striped snakehead and horseface loach compared to their respective total sum of EPA and DHA content. Though, the highest ARA content amongst the seven Bukit Merah species was recorded in the beardless barb and also Javanese barb. The high content of ARA in the striped snakehead fish has been a highlighted feature, as ARA is a precursor for prostaglandins which plays a major role in wound healing (Baie & Sheikh 2000). Striped snakeheads are revered as an important food to be consumed for its medicinal properties by certain members of the community here in Malaysia (Mohd & Abdul Manan 2012).

Sum of EPA and DHA has been recommended to be used as an indicator of nutritive value of fish for humans (Kris-Etherton *et al.* 2009). Fig. 2 indicates that tinfoil barb and Javanese barb has the highest EPA+DHA comparatively which suggests higher nutritive value to the rest of the species. The least EPA+DHA content was found to be in the muscle of the striped snakehead. No significant differences were observed in the n-3/n-6 ratio amongst all of the species analysed as well. The imbalance of n-3/n-6 ratios in human nutrition has been highlighted with the rise in consumption of lower omega-3 fatty acid rich foods which could lead to poor health conditions in certain individuals (Strobel *et al.* 2012).

Feeding habits and availability of organisms in the freshwater food web affects the fatty acid composition of individual fish regardless of species. Fatty acid deposition in muscle of fish is also dependent on the possession of the PUFA conversion enzymes of the organism (Tocher 2003). Of two piscivorous species, the hampala barb and the striped snakehead, they seem to reflect different patterns in some of the fatty acid components. For instance, as mentioned, striped snakehead showed the lowest amount of EPA and DHA, however this was not seen in the hampala barb. The differences were not statistically significant due to the high standard error obtained in the analysis of the hampala barb. This could be due to the varying food which was consumed by each individual fish which contributed to its vast difference amongst replicates. This was unavoidable and is reported as true obtained data.

The perception from the consumer is also of importance when it comes to selecting fish for purchase. In a review on consumer fish and seafood purchasing behaviour in several developed countries, the driving factors and also barriers towards eating fish were identified (Carlucci *et al.* 2015). Amongst the factors, the country of fish origin, preserving methods, packaging and personal values played significant roles in fish consumption. Consumers tended to show concerns about the distance of fish production which alters the utilisation of preservation treatments. Locally caught fish were preferred as they required less preservation, and reduced transport cost (Birch & Lawley 2012). Majority of consumers also perceive wild caught fish to be better compared to farmed fish in terms of flavour/taste, safety, nutritional and health value (Carlucci *et al.* 2015). The preference and fish purchasing behaviour of the community which has access to the fish of Bukit Merah Reservoir could not be evaluated in further detail as a study as such has yet to be conducted in this area. It was also revealed that the older generation seemed to have a more positive attitude towards eating fish. Alongside this group, those who are well educated and have a better understanding towards the nutrient contents in fish tend to lean more towards including fish in their diets (Olsen 2003).

The objective of this study was to investigate the nutritional value of freshwater fish in the Bukit Merah Reservoir. Based on this initial fatty acid profiling in muscle of selected fish species from the said reservoir, it can be concluded that in general, the cyprinid fish contain substantial amount of omega-3 LC-PUFA compared to members from family Channidae and Cobitidae. Tinfoil barb and Javanese barb recorded the highest levels of combined EPA and DHA indicating the highest nutritional value comparatively. Even though it was expected that fish in the higher trophic level such as striped snakehead to contain higher levels of LC-PUFA compared to the detritus/plant eating fishes, it was not reflected in this study. The omega-3 PUFA content of fish varies depending on the fish species and is affected by their feeding habits. Predominantly, the evidence of health benefits is usually associated with marine sourced omega-3 LC-PUFA, with a lack of consumer understanding in alternative omega-3 sources. It is recommended that consumers should be better informed that a consumption of diet that included moderate levels of seafood within a balanced diet is the best way to obtain the omega- 3 LC-PUFA related health benefits (McManus *et al.* 2011). According to a study comparing marine and freshwater fish species conducted in Turkey, even though the omega-3 PUFA of marine fish were higher than those of freshwater fish, most of the freshwater fish were primarily comparable to those of marine fish as sources of PUFA. They deduced that both marine and freshwater fish were capable of being good supply of EPA and DHA (Özogul *et al.* 2007). This also goes to show that consuming any one type of fish does not necessarily fulfil LC-PUFA requirements in humans. In order to maintain a wholesome health condition, it is recommended that consumption of fish be widened to varieties of different species not only constricting to either fish from marine or freshwater habitats.

## Figures and Tables

**Table 1:** Selected edible fish species identified from Bukit Merah Reservoir

Family	Species	Common Name	Local Name
Cyprinidae	*Barbonymus schwanenfeldii*	Tinfoil barb	Lampam sungai
	*Barbonymus gonionotus*	Javanese barb	Lampam Jawa
	*Hampala macrolepidota*	Hampala barb	Sebarau
	*Cyclocheilichthys apogon*	Beardless barb	Temperas
	*Oxygaster anomalura*	Glassfish	Lalang
Channidae	*Channa striata*	Striped snakehead	Haruan
Cobitidae	*Acantopsis dialuzona*	Horseface loach	Pasir

**Table 2:** Fatty acid (% of total fatty acids) profile in muscle of edible fish species from Bukit Merah Reservoir.

Fatty acid	Tinfoil barb	Javanese barb	Hampala barb	Beardless barb	Glassfish	Striped snakehead	Horseface loach
14:0	0.6 ± 0.2	1.2 ± 0.3	2.3 ± 0.5	0.6 ± 0.0	1.2 ± 0.3	1.5 ± 0.3	2.2 ± 1.8
16:0	24.8 ± 2.3	21.9 ± 1.0	27.2 ± 1.3	21.5 ± 0.3	23.2 ± 0.7	23.3 ± 0.3	25.5 ± 11.4
18:0	12.9 ± 0.2	13.4 ± 0.9	9.5 ± 0.7	12.0 ± 0.2	12.1 ± 0.4	9.8 ± 0.6	12.1 ± 3.9
∑SFA	40.1 ± 2.5	41.1 ± 1.0	40.6 ± 0.4	40.0 ± 0.3	38.8 ± 0.2	38.4 ± 0.8	43.1 ± 18.6

16:1	1.3 ± 0.1	3.04 ± 0.4	6.6 ± 1.9	2.8 ± 0.1	2.9 ± 0.6	3.1 ± 0.3	5.0 ± 2.8
18:1n9	15.5 ± 3.6	7.6 ± 1.3	13.3 ± 5.2	9.5 ± 0.6	21.0 ± 1.2	23.2 ± 1.0	13.7 ± 8.4
18:1n7	2.6 ± 0.4	2.2 ± 0.3	3.4 ± 0.8	3.8 ± 0.3	2.8 ± 0.4	4.0 ± 0.5	3.3 ± 1.8
∑MUFA	23.2 ± 2.7	16.5 ± 1.3	23.9 ± 2.6	16.2 ± 0.4	28.1 ± 2.2	34.2 ± 1.7	24.0 ± 13.0

18:3n3	0.9 ± 0.1	1.4 ± 0.2	4.3 ± 3.9	0.9 ± 0.1	1.3 ± 0.2	2.7 ± 0.7	1.6 ± 1.3
18:4n3	0.2 ± 0.0^ab^	0^a^	0.3 ± 0.2^ab^	0.5 ± 0.1^b^	0.1 ± 0.0^a^	0.6 ± 0.1^b^	0^a^
20:3n3	0.7 ± 0.0	0.7 ± 0.0	1.4 ± 0.8	1.2 ± 0.0	1.0 ± 0.0	0.8 ± 0.3	1.9 ± 0.4
20:4n3	0.2 ± 0.0	0.3 ± 0.0	0.4 ± 0.3	0.3 ± 0.0	0.2 ± 0.1	0.1 ± 0.0	0
20:5n3	2.1 ± 0.5	3.9 ± 0.3	3.0 ± 2.3	2.4 ± 0.2	2.1 ± 0.0	0.9 ± 0.2	1.9 ± 0.3
22:5n3	1.4 ± 0.3	2.3 ± 0.1	1.9 ± 1.0	2.8 ± 0.1	1.2 ± 0.0	2.2 ± 0.4	2.8 ± 0.4
22:6n3	12.2 ± 2.4^b^	13.1 ± 0.6^b^	9.3 ± 0.7^ab^	10.3 ± 0.2^ab^	10.6 ± 1.1^b^	4.0 ± 0.9^a^	8.4 ± 1.7^ab^
∑n-3	17.6 ± 3.0	21.7 ± 0.5	20.7 ± 7.5	18.4 ± 0.4	16.6 ± 0.8	11.3 ± 0.2	16.6 ± 3.9

18:2n6	8.9 ± 0.8	4.9 ± 0.6	9.0 ± 4.5	7.5 ± 0.1	6.2 ± 0.6	8.7 ± 1.1	10.0 ± 5.3
18:3n6	0^a^	0^a^	0.31 ± 0.1^cd^	0.2 ± 0.1^abc^	0.3 ± 0.0^bcd^	0.5 ± 0.1^d^	0^a^
20:3n6	0.1 ± 0.0^a^	0.1 ± 0.0^a^	0.1 ± 0.0^a^	0.2 ± 0.0^a^	0.2 ± 0.0^a^	0.4 ± 0.1^b^	0^a^
20:4n6	9.8 ± 1.6^ab^	15.7 ± 1.7^bc^	5.5 ± 0.6^a^	17.6 ± 0.4^c^	9.7 ± 1.4^ab^	6.0 ± 1.3^a^	10.8 ± 1.6^abc^
∑n-6	18.8 ± 2.2	20.7 ± 1.2	14.8 ± 5.3	25.5 ± 0.5	16.3 ± 1.8	15.5 ± 2.4	20.9 ± 6.1
∑PUFA	36.4 ± 4.8	42.4 ± 1.7	35.5 ± 2.3	43.9 ± 0.6	32.8 ± 2.2	26.8 ± 2.4	37.5 ± 10.0
n-3/n-6	0.9 ± 0.1	1.1 ± 0.0	1.8 ± 1.2	0.7 ± 0.0	1.0 ± 0.1	0.8 ± 0.1	0.8 ± 0.0
EPA+DHA	14.3 ± 2.7^b^	17.0 ± 0.5^b^	12.3 ± 3.0^ab^	12.7 ± 0.3^ab^	12.7 ± 1.1^ab^	4.9 ± 0.7^a^	10.3 ± 2.0^ab^

*Notes*: Mean values in similar row with different superscript letters are significantly different (Tukey’s HSD, *p* < 0.05)
